# Case report: A rare case of *TBL1XR1-RARB* positive acute promyelocytic leukemia in child and review of the literature

**DOI:** 10.3389/fonc.2022.1028089

**Published:** 2022-11-16

**Authors:** Mingyan Jiang, Jinrong Li, Jianrong Wu, Yiping Zhu, Ju Gao

**Affiliations:** ^1^ Department of Pediatrics, West China Second University Hospital, Sichuan University, Chengdu, China; ^2^ Key Laboratory Of Birth Defects And Related Diseases Of Women And Children (Sichuan University), Ministry Of Education, Chengdu, China

**Keywords:** acute promyelocytic leukemia, TBL1XR1-RARB fusion, ATRA resistance, prognosis, literature review

## Abstract

Some forms of acute myelogenous leukemia (AML) share typical morphological and immunophenotypic features of acute promyelocytic leukemia (APL) but are negative for promyelocytic leukemia-retinoic acid receptor alpha (*PML-RARA*) fusion. These forms of AML are known as variant APL. Some variants of APL present with retinoic acid receptor beta (*RARB*) fused or rearranged with partner genes. *RARB*-positive APL is very rare, resistant to all-trans retinoic acid (ATRA), and associated with poor prognosis. Here, we reported one case with *TBL1XR1-RARB* positive APL, featured by early onset and no apparent bleeding tendency or coagulation dysfunction. This patient was resistant to ATRA and arsenic trioxide (ATO), but was good responsive to conventional chemotherapy for AML. The case report was followed by a literature review.

## Introduction

Acute promyelocytic leukemia (APL) is a special type of acute myeloid leukemia (AML), presenting with an abnormal increase in promyelocytes in bone marrow and peripheral blood (1). Patients with APL are usually combined with apparent coagulation disorders. Most of the previously reported APL patients die at an early age. Currently, chemotherapy with all-trans retinoic acid (ATRA) plus arsenic trioxide (ATO) can dramatically improve the prognosis of APL patients (2). The majority of APL patients are positive for the *PML-RARA* fusion, which is caused by t(15;17) translocation, resulting in the sensitivity to ATRA (3). However, very few patients have typical morphological and immunophenotypic characteristics of APL (e.g., promyelocytes containing coarse azurophile granules and Auer bodies) but are negative for promyelocytic leukemia-retinoic acid receptor alpha (*PML-RARA*) fusion. These forms of AML are known as variant APL. Some variants of APL present with retinoic acid receptor beta (*RARB*) rearrangement (4). *RARB*, *RARA*, and retinoic acid receptor gamma (*RARG*) are all members of the nuclear receptor superfamily and share high homology (90%). Nearly all variants of APL are resistant to ATRA and associated with a poor prognosis (5). Here we reported one rare case with *TBL1XR1-RARB*-positive variant of APL. This patient had an early onset, high white blood cell count upon initial visit, no apparent bleeding tendency or coagulation function. Besides, this patient was resistant to ATRA, but good responsive to the conventional chemotherapy for AML.

## Case presentation

The female patient, aged 2 years and 1 month, was admitted to the Department of Pediatric Hematology and Oncology at West China Second University Hospital of Sichuan University on September 8, 2021 due to ecchymosis, pale and sallow complexion for one month, and fever for one day. This patient had no joint swelling and pain, epistaxis, hematuria, convulsions, jaundice, pallor, cough, or diarrhea during the course of disease. Her mother was of the Hui ethnic group. Family history of hematological malignancies or hereditary disorders was denied. No significant abnormalities were reported during pregnancy or at the growth and development stage of the pediatric patient.

Physical examinations upon admission: T 36.5°C, P 118/min, BP 88/57mmHg, R 26/min, height 84 cm, and weight 10.7 kg. The patient was with a few pinpoint bleeding spots and petechia, but without mass ecchymosis and hematoma. Superficial lymph nodes were not enlarged. The liver was palpable at 2.5 cm below the costal margin and at 3.5 cm below the xiphoid process. The spleen was not palpable below the costal margin.

**Figure 1:**
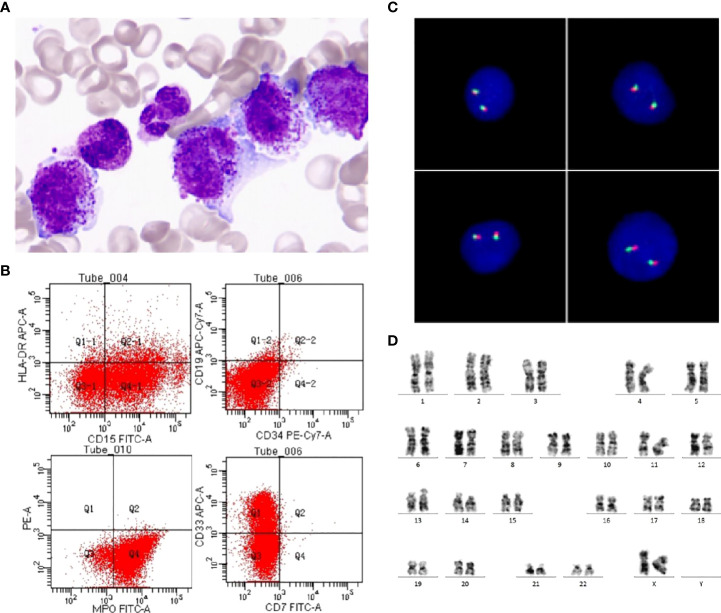
Morphology, immunophenotyping, FISH and karyotyping. **(A)** An increased number of promyelocytes, which contained azurophilic granules or Auer bodies (Wright-Giemsa staining of bone marrow, ×1000). **(B)** Leukemia cells expressed MPO, CD13, CD15, CD33, but do not express CD34, HLA-DR. **(C)** No *PML-RARA* fusion signals were detected by FISH using *PML-RARA* Dual Color, Dual Fusion Translocation Probe. **(D)** Chromosome G band analysis revealing karyotype 46,XX [20].

Routine blood test upon initial visit: white blood cell count 41.0×10^9^/L, neutrophil count 17.0×10^9^/L, percentage of juvenile cells 13.00%, hemoglobin level 74g/L, platelet count 29×10^9^/L, and C-reactive protein (CRP) 1.9 mg/L. Coagulation function screening: FDP 25.97ug/ml (normal range<5 µg/mL), D-dimer 12.41 mg/L (normal range <0.55 mg/L). Blood biochemistry test: LDH 1621U/L, no other abnormalities were found. Bone marrow smear: Positive for myeloperoxidase (MPO) staining, pathological promyelocytes accounting for 82%, with coarse azurophilic granules densely distributed in the cytoplasma and fagot Auer bodies present, hypergranular APL (M3) diagnosed (**Figure 1A**). Immunophenotyping: AML (expressing CD13, CD15, CD33, CD64 and MPO, but no HLA-DR, CD34, CD19 or other antigens recognized by T-cells and B-cells) (**Figure 1B**). Immunophenotyping performed by Hystou Technology Co., Ltd.: Abnormal cells seen where myeloid cells extended towards primitive cells, accounting for about 85% of karyocytes and primarily expressing CD9, CD13, CD15, CD33, CD38, CD58, CD64, CD123, and MPO, with some expressing CD56, not expressing CD34 and HLA-DR. *RARA* rearrangement or *MLL* rearrangement was not identified by fluorescence *in situ* hybridization (FISH) (**Figure 1C**). The patient was found with karyotype 46, XX [20] (**Figure 1D**) We did the *PML-RARalpha* fusion gene test (using polymerase chain reaction, PCR and FISH methods) once we suspected APL of the patient on day 1, and re-checked the results of *PML-RARalph*a in two different medical laboratory institutions. Till then, we confirmed the negative of *PML-RARalpha*. Besides, the patient received detections for 67 mutations associated with myeloid hematologic diseases: *EZH2* c.1958A>G p.Gln653Arg. Epstein-Barr virus DNA, cytomegalovirus DNA, purified protein derivative test and blood culture were negative. The patient had normal humoral immunity, autoantibody test, G6PD, ferritin, cardiac uhrasonography, electrocardiography, and head CT. Thoracic and abdominal CT scans: mild inflammations in bilateral lungs, with slightly thickened dorsal pleura on the two sides; slightly enlarged liver, with multiple enlarged lymph nodes near the abdominal aorta and at the root of mesentery.

Treatment process: The patient was preliminarily diagnosed with APL based on her clinical manifestations, bone marrow morphology, and immunophenotypic profile. On the day of admission (September 7, 2021), the patient received induction chemotherapy using ATRA and ATO for high-risk APL to induce the differentiation and apoptosis of APL cells. Hydroxycarbamide and cytarabine were prescribed as chemotherapy medication. Dexamethasone was given to prevent differentiation syndrome, piperacillin/tazobactam was given as an anti-infective regimen, and micafungin was given as the antifungal agent. Allopurinol was used to prevent hyperuricemia. Otherwise, the patient had no signs of cell differentiation, including fever, polyserous effusion, increased white blood cell count, and increased percentages of myelocytes and metamyelocytes. And the coagulation disorder was non-significantly abnormal. So we speculated variant APL caused by some rare fusion genes and did whole transcriptome resequencing on day 8. Meanwhile, we were not sure whether the variant APL was sensitive to ATRA and ATO, so we continued the ATRA-ATO therapy.

The patient was reassessed by bone marrow smear on day 28 of chemotherapy (October 5), which showed that promyelocytes accounted for 6%, with minimal residual disease (MRD) <0.01%. Ribozero’s method was used to remove ribosomal RNA from total RNA, and then reverse transcribed it into cDNA. cDNA was used as a template to construct a library supporting sequencing. Using Illumina Hiseq X sequencing platform to perform full transcriptome level detection of RNA in the samples of subjects, gene fusion and SNVs/Indels variation at the RNA level can be analyzed. The total amount of data obtained by this sequencing is 16.64Gb. The sequenced fragments were compared with EnsemblGRCh37 reference genome through STAR software. Varscan software was used for mutation detection. Arriba was used to predict gene fusion. The mutation results were annotated with annovar for downstream data. The annotation databases mainly included Clinvar, dbSNP, 1000 genome, genomeAD, ExAC, COSMIC. Fusion gene database included COSMIC, Fusion cancer, Atlas of Genetics and Cytogenetics in Oncology and Haematology, My Cancer Genome. On day 36 of chemotherapy (October 13), the result of whole transcriptome resequencing revealed the following: *TBL1XR1-RARB* fusion gene and *TBL1XR1* mutation (NM-024665: exon 5: c.423-424insTA: p.A142*).

The treatment was adjusted to the induction chemotherapy DAH for AML according to the Chinese Children Leukemia Group protocol (CCLG-AML-2019), consisting of daunorubicin (DNR), cytarabine (Ara-C), and homoharringtonine (HHT). On day 64 of chemotherapy (November 10), the patient was reassessed by bone marrow smear, which showed that promyelocytes accounted for 4.5%, indicative of complete remission. And *TBL1XR1-RARB fusion gene and TBL1XR1* mutation were negative, with MRD<0.01% (**Table 1**). Then she received the second induction regimen IAH, consisting of idarubicin (IDA), Ara-C and HHT, and four consolidation regimens, including MA (mitoxantrone and Ara-C), HA (HHT and Ara-C), CLASP (Ara-C and L-asparaginase) and HAs (HHT and Ara-C). In the meanwhile, the patient was continuous complete remission (CCR) monitored by bone marrow smear, *TBL1XR1-RARB fusion gene* and MRD. Her parents refused to receive hematopoietic stem cell transplantation (HSCT) although she was matched 12/12 in China Marror Donor Program (CMDP). After the consolidation therapy, she received the maintenance therapy, including Ara-C (50mg/m^2^/d) and 6-mereaptopurine (6-MP, 40mg/m^2^/d, d1-d4, q4w). Until now, the patient was still CCR and was being followed up.

**Table 1:** Blood cell counts, percentage of promyelocytes upon bone marrow smear, bone marrow MRD by flow cytometry, FISH, and qPCR detection of *TBL1XR1-RARB* fusion during the course of treatment.

Treatment day	Day 1	Day 14	Day 28	D36	Day 64
WBC (×10^9^/L)	41.0	3.2	3.8	1.7	2.8
HGB (g/L)	65	80	85	98	103
PLT (×10^9^/L)	29	33	535	320	740
PT (s)	13.5	12.4	13.7	13.7	13.0
APTT (s)	25.9	26.8	28.2	39.0	28.5
Fg (mg/dL)	250	217	254	100	246
D-Dimer (mg/L)	12.41	0.78	0.79	1.09	0.24
FDP (μg/mL)	32.43	2.8	3.87	4.11	1.28
ATIII (%)	106	111	93	51	102
BM smear(%)	82.0	3.0	6.0	–	4.5
BM FC MRD	–	–	<10^-4^	–	<10^-4^
FISH	–	–	–	–	neg
qPCR	–	–	–	–	neg

WBC, white blood celll; HGB, hemoglobin; PLT, platelet; PT, prothrombin time; APTT, activated partial thromboplastin time; Fg, fibrinogen; FDP, fibrin degradation products; ATIII, antibhrombin III; BM FC, bone marrow flow cytometry; MRD, minimal residual disease; FISH, fluorescent in situ hybridization; qPCR, quantitative polymerase chain reaction. The level of TBL1XR1-RARB transcripts were normalized to the reference gene ABL1 as the normalized copy number (based on the ΔC_p_ method).

## Discussion

Acute promyelocytic leukemia (APL) is a unique subtype of acute myeloid leukemia (AML), which is characterized by an increase in promyelocytes in the bone marrow (1). About 95% of APL patients are positive for *PML-RARA* fusion and should start the ATRA treatment as early as possible if APL is suspected. The prognosis of APL has been improved dramatically in recent years due to the widespread use of ATRA and ATO. APL has become the subtype of AML with the highest cure rate, the long-term survival rate being 95% and above (2). However, 5% of the APL patients are of the refractory/recurrent type. These APL patients are usually of high-risk classic APL or variant APL. The latter is usually negative for *PML-RARA*, resulting in unresponsiveness to ATRA and high mortality. *TBL1XR1-RARB* is a fusion that occurs in fewer variants of APL (4). Early recognition of this fusion gene can inform the decision about treatment strategies and improve the prognosis. It is generally believed that the combination chemotherapy using ATRA and ATO can improve the prognosis of high-risk classic APL. However, recognizing, diagnosing and treating variant APL still remains a challenge. According to the latest opinion, variant APL collectively refers to *PML-RARA*-negative APL, including APL with *RARA*, *RARB* and *RARG* rearrangements and other genetic abnormalities (4). So far, we are still uncertain about the pathogenesis and the choices of clinical treatments for variant APL.

Interestingly, the MRD result at the end of ATO-ATRA cycle was the same as it after DAH. We speculated that the reasons were: First of all, there were differences in the methods of detecting MRD of AML, and the techniques were not standardized, which would result into significant fluctuation of results. Secondly, we speculated that the reduction of residual disease were possibly caused by the use of cytarbine rather than ATRA and ATO. Thirdly, the partial remission of bone marrow smear on day 28 and no signs of cell differentiation supported the speculation. Besides, we did the fusion gene test once we suspected APL of the patient, and re-checked the results of *PML-RARalph*a in two different medical laboratory institutions. Till then, we confirmed the negative of *PML-RARalpha*. Without *PML-RARalph*a, we doubted the diagnosis. But the bone marrow smear and Immunophenotyping were very typical, so we speculated that there maybe some rare fusion genes caused a variant APL and did RNA-Seq further. Meanwhile, we were not sure whether the variant APL was sensitive to ATRA and ATO, so we continued the ATRO-ATO therapy until we got the result of RNA-Seq. As mentioned in the references, once we suspected APL, we should start ATRA-ATO therapy because most of the APL was caused by *PML-RARalpha*. Meanwhile, once we confirmed the negative result of *PML-RARalph*a and positive of *TBL1XR1-RARB*, we adjusted the chemotherapy to induction therapy of AML. So we would like to emphasize the importance of RNA-Seq.

So far, six *TBL1XR1-RARB*-positive variant APL patients have been reported (**Table 2**). The pediatric case reported in this study had typical morphological and Immunophenotypic features of APL. This patient had an early onset and insignificant bleeding tendency and coagulation disorders. Besides, the patient was unresponsive to ATRA. The above clinical manifestations agreed well with those from the previously reported cases. That is, *RARA*-negative APL patients had higher white blood cell count and platelet count at the acute stage than *PML-RARA*-positive patients. The former were resistant to ATRA and more likely to experience failure of induced differentiation of APL cells (4, 5). RNA-Seq technology provides important diagnostic information for APL with atypical clinical manifestations but typical morphological and immunophenotypic profile. Nevertheless, morphological features provide important diagnostic clues for APL diagnosis. Most of the six reported cases of *TBL1XR1-RARB*-positive APL are young children aged 2.9 years old on average. These pediatric cases of APL might have unique genetic background. *RARB* translocation may be a genetic marker of juvenile APL patients. It is necessary to include elder and adult APL patients with *RARB* translocation to obtain more accurate data on translocation frequency, clinical features, and treatment outcomes (6). Besides, two patients with *TBL1XR1-RARB*-positive APL had extramedullary recurrence, a rare condition in classic APL. Five out of six cases with *TBL1XR1-RARB*-positive APL were resistant to ATRA. Two of the resistant cases were also unresponsive to ATO. The general features of the previously reported patients were consistent with those of our case. Moreover, patients with *TBL1XR1-RARB*-positive APL were also resistant to some other chemotherapeutic agents. The conventional chemotherapy regimens for AML are the most commonly used to treat *TBL1XR1-RARB*-positive APL, though the failure of achieving a remission or experiencing early recurrence increases. Tamibarotene, with proven efficacy for recurrent APL, is found ineffective for *RARB*+ cells, either *in vivo* or *in vitro* (6). Therefore, it is urgent to develop a novel small molecule complex for precision treatment of APL with RARB translocation.

**Table 2:** variant APL with *TBL1XR1-RARB* fusion in literatures.

No.	Age at diagnosis (years)/sex	WBC (×10^9^/L)	Morphology	Immunophenotype	Cytogenetics	FDP level at diagnosis (μg/mL)	Other related gene mutations	Response to ATRA	Events (Site of relapse)	HSCT (Disease status in HSCT)	Time to relapse from diagnosis	Outcome (Follow-up period)	Reference
1	2.6/M	53.1	promyelocytes with azurophilic granules, few faggot cells	CD13+ CD33+ Cy-MPO+ CD24_ CD58+ CD99+ CD244+ CD34- HLA-DR-	46,XY,t(3;10;12)(q26.2;q22;q15)[20/20]	20.1	None	None	Relapse (bone marrow and extramedurally)	Yes (2CR)	0.6 years	Alive in 2CR (2.5 years)	(4)
2	4.3/F	6.1	promyelocytes with azurophilic granules, few faggot cells	CD13+ CD33+ CD99+ Cy-MPO+ CD15+ CD65+ CD34- HLA-DR-	46,XX,del(2)(p)?,inv(4)(p16q12)[1/20],45,idem,-X[3/20],46,idem,del(3)(p25)[3/20],46,XX[13/20]	503	None	None	Induction failure	Yes (1CR)	Induction failure	Alive in 1CR (6.1 years)	(4)
3	4.1/F	14.9	promyelocytes with azurophilic granules, few faggot cells	CD13+ CD33+ Cy-MPO+ CD15+ CD64+ CD65+ CD34- HLA-DR-	47,XX,+3[19/20]	110.6	None	None	Relapse (bone marrow)	Yes (2CR)	1.1 years	Alive in 2CR (7.2 years)	(4)
4	0.9/F	30.5	NA	NA	47,XX,+6 (2)/46,XX	NA	None	NA	Relapse	Yes	Relapse	Alive	(5)
5	0.98/M	128.3	NA	NA	46,XY	NA	None	Yes	None	None	None	Alive in 1CR (9 years)	(3)
6	4.76/M	24.3	NA	NA	46,XY	NA	None	Yes	Relapse (neck lymph node and BM)	None	7 months and 22 months	Death in 2CR (30 months)	(3)
7	2/F	41.0	promyelocytes with azurophilic granules, few faggot cells	CD13+ CD15+ CD33+ CD64+ MPO+, HLA-DR- CD34- CD19-	46,XX	32.43	EZH2 c.1958A>G p.Gln653Arg, *TBL1XR1* exon 5: c.423-424insTA: p.A142*	None	None	None	None	Alive	Present study

ATRA, all-trans retinoic acid; ATO, arsenic trioxid; NA, not applicable.

**Figure 2:**
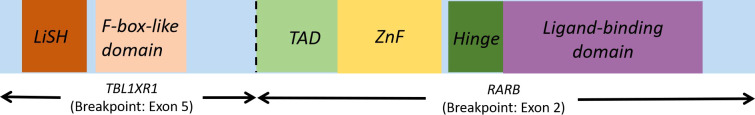
*TBL1XR1-RARB fusion gene.* Lish, Lis homology domain TAD, transcription activation domain, ZnF, Zn finger domain.

*RARB* is located at 3p24. Only one *RARB* fusion gene (*TBL1XR1-RARB*) has been identified in variant APL. It has been found that *TBL1XR1* is the only partner of RARA in variant APL (7). *TBL1XR1* is located at 3q26, and pediatric patients with APL usually have t(3;3) or inv(3) (**Figure 2**). *RARB* and *RARA* perform similar functions and both are members of the family of retinoic acid receptors (RARs). *RARB* and retinoid X receptor (RXR) form heterodimers, which further bind to RAREs. RARs/RXR heterodimers can recruit co-inhibitory factors and histone deacetylase by targeting the genes in quiescent cells. RARs/RXR heterodimers can be activated by physiological concentrations of ATRA, thereby mediating embryogenesis, cell growth and differentiation. As analyzed above, the RAS pathway plays an important role in the pathogenesis of APL. *TBL1XR1-RARB* fusion is the recurrent genetic abnormality in APL. *TBL1XR1* and *RARB* can form a homodimer, which exhibits a significant negative effect on *RARA* and *RARB*. This will result in the arrest of promyelocyte differentiation and maturation and hence promote continuous cell proliferation (6). However, basic researches have shown that ATRA has a much weaker impact on *TBL1XR1-RARB* than on *PML-RARA*. *TBL1XR1-RARB*-positive cells are resistant to ATRA, ATO and tamibarotene (4–6, 8).

Besides, our patient was also combined with mutations in the *TBL1XR1* and *EZH2* genes. The A142* mutation in the *TBL1XR1* gene is a nonsense mutation. Somatic mutations in the *TBL1XR1* gene are mostly loss of function mutations, which usually occur in lymphoma, but rarely in AML. *TBL1XR1*, also known as *TBLR1*, encodes for a transcriptional regulatory protein, which belongs to the family of WD40-repeat (WDR) proteins (9). *TBL1XR1* binds to the NCoR (nuclear receptor corepressor)/SMRT (silencing mediator of retinoic acid and thyroid hormone receptors) complex, acting as a transcriptional corepressor that mediates deacetylation of targeted genomic proteins (9). *TBL1XR1* works together with *TBL1X* to stabilize the NCoR/SMRT complexes on the chromatin by interacting with histones H2B and H4 (10). In addition, *TBL1XR1* mediates the ubiquitination and degradation of the NCoR/SMRT complexes after ligand binding to nuclear receptors (11). Ligand binding activates several hormone receptors, including androgen receptors and retinoic acid receptors (12). *TBL1XR1* is involved in the regulation of both the NF-κB and the WNT signaling pathways (9). *RARB*-positive APL with mutations in the *TBL1XR1* gene has been reported, indicating that *TBL1XR1* is also involved in APL (13). Similar to our pediatric patient, the previously reported cases were not found to combine with *FLT3-ITD* mutation, a common mutation in adult patients with APL. In addition, EZH2 c.1958A>G p.Gln653Arg is a member of the PRC2 family, encoding for histone methyltransferase. Simple *EZH2* mutations have no impact on overall survival (OS) or progression-free survival (PFS) (13). However, *EZH2* mutation combined with *FLT3* or *IDH2* mutation usually indicates poor OS and PFS.

Overall, as shown by the present case, APL with typical morphological and immunophenotypic characteristics but atypical clinical manifestations may be *PML-RARA*-negative APL. RNA-Seq is of high importance to confirm the diagnosis of APL and to identify other fusion genes with prognostic impact. *TBL1XR1-RARB*-positive APL is usually associated with poor prognosis and resistance to ATRA. Such patients may be treated by the conventional chemotherapy for AML, but more aggressive treatments, such as allogeneic hematopoietic stem cell transplantation, are recommended. However, our findings need to be confirmed by further investigations.

## Data availability statement

The original contributions presented in the study are included in the article/supplementary material. Further inquiries can be directed to the corresponding author.

## Ethics statement

The studies involving human participants were reviewed and approved by West China Second University Hospital, Sichuan University. Written informed consent to participate in this study was provided by the participants’ legal guardian/next of kin.

## Author contributions

MJ and JL designed the experiments. Jw and JG performed the experiments. MJ, YZ and JL collected and analyzed the data. JG drafted manuscript. All authors contributed to the article and approved the submitted version.

## Funding

1.Youth Innovation Project of Medical Research in Sichuan Province (Q18015). 2.Application Foundation Program of Science and Technology Department of Sichuan Province(2021YJ0453). 3.Application Foundation Program of Science and Technology Department of Sichuan Province (2020YFS0253).

## Conflict of interest

The authors declare that the research was conducted in the absence of any commercial or financial relationships that could be construed as a potential conflict of interest.

## Publisher’s note

All claims expressed in this article are solely those of the authors and do not necessarily represent those of their affiliated organizations, or those of the publisher, the editors and the reviewers. Any product that may be evaluated in this article, or claim that may be made by its manufacturer, is not guaranteed or endorsed by the publisher.
